# Modernising physician resource planning: a national interactive web platform for Canadian medical trainees

**DOI:** 10.1186/s12913-021-07366-4

**Published:** 2022-01-27

**Authors:** D. Bourcier, B. W. Collins, S. M. Tanya, M. Basu, A. P. Sayal, S. Moolla, A. Dong, M. Balas, H. Molcak, G. Punchhi

**Affiliations:** 1 Dalhousie University, Halifax, Nova Scotia Canada; 2 Memorial University of Newfoundland, St. John’s, Newfoundland and Labrador Canada; 3 University of Toronto, Toronto, Ontario Canada; 4 McMaster University, Hamilton, Ontario Canada; 5 University of British Columbia, Vancouver, British Columbia Canada; 6 Western University, London, Ontario Canada

**Keywords:** Physician resource planning, Physician workforce, Health human resources, Career planning, Medical school

## Abstract

**Background:**

Healthcare systems rely heavily upon human resources to ensure high-quality access to care for the general population. With significant health worker shortages predicted worldwide in the coming decades, maximizing the current workforce by means of a physician resource planning (PRP) strategy that ensures the right number, mix, and distribution of physicians to meet population needs is warranted. In Canada, there is an insufficient number of primary care providers, and disproportionately low numbers of specialist physicians in rural compared to urban regions. Currently, Canadian medical students are not effectively included in PRP strategy and lack the required information for career orientation to help rebalance the population’s workforce needs. This paper present the *Health Human Resource (HHR) Platform*, a comprehensive web tool that includes relevant workforce data to empower medical students in choosing a discipline based on both personal interests and social accountability.

**Results:**

Physician workforce data, comments from Canadian residency program directors, and career planning resources were collected by the Canadian Federation of Medical Student’s (CFMS) HHR Task Force. This information was consolidated to create a national interactive platform that uses a map, comparison table, and trend graphs to illustrate over 500,000 unique data points from 37 datasets, including specific information and resources spanning 62 medical specialties from 2015 onwards. There was a 24.6% response rate for program director comments. During the first 4 months of the *HHR Platform* launch, there were 2434 different users, of which 985 were returning, with an average of 20.0 users per day spending on average 3 min on the platform.

**Conclusions:**

The *HHR Platform* constitutes a national approach to PRP informing medical students on the mix and distribution of physicians needed to meet the future healthcare demands of the Canadian population.

## Background

Healthcare accounts for a significant portion of public expenditure in many countries globally, and human resources occupy a majority of this spending [1]. Imbalances in healthcare workforce supply and demand negatively impact the provision of health services and ultimately strain overall public expenditure [2–4]. Even under universal healthcare systems, such as the Canadian framework, these imbalances result in adverse sequelae. For example, between 2005 and 2019, the physician to 100,000 population ratio increased from about 190 to 240, representing one of the greatest rises of physician supply in Canadian history, while the proportion of Canadians without a family physician increased from 13.6 to 14.5% during this period [5–7]. Ultimately, patient morbidity and mortality are known to rise when the supply of healthcare providers does not match population needs [8–10]. This mismatch may be attributed to decades of overtraining subspecialist physicians, which has resulted in undersupply of primary care specialists [11, 12]. Furthermore, there is a geographic dichotomy: only 10% of the total physician workforce and only 2% of specialized physicians practice in rural Canada where 18% of the population is situated [13]. Therefore, strategies to improve patients’ access to care in Canada are necessary.

To address this, Health Canada invested $1.8 million in 2010 to review postgraduate medical education in Canada [14]. One of their recommendations was to iteratively re-evaluate medical training programs to ensure the right “physician mix, distribution, and number” of generalist and specialist positions to serve the Canadian population. At a national level, Canadian medical residency training has historically seen a deficit of students applying to primary care programs resulting in unfilled training positions; for example there were 169 vacant family medicine positions in 2020 [15]. This may be due to the perception that it is not a prestigious specialty and lacks innovative technology or academic opportunities [16, 17] In contrast, a 2019 survey of all the specialities and subspecialities offered in Canada showed that 34% of the total physicians surveyed were unable to secure employment at 12-17 months following graduation [18].

Health human resource (HHR) planning is a necessary endeavour to optimize the quantity and skillset of our healthcare workforce, which in turn ensures that population health needs are adequately serviced. It is the most sustainable approach to attaining long-term stability of the Canadian universal healthcare system [2]. Physician resource planning (PRP), a subset of HHR strategy, aims to optimize physicians’ available supply to meet population demands. Many nations, including Canada, are predicted to face critical deficits in physician supply over the next 30 years [19]. The goal is not to simply train a set number of physicians, but rather to train the right types of physicians based on population needs. This avoids the present circumstance of simultaneously having too many physicians, and yet, not enough. These shortages may be effectively overcome by improving PRP strategies for domestically trained physicians to close gaps in attrition and supply-demand mismatch [11]. However, due to the paucity of concerted national PRP efforts, Canadian medical trainees are not equipped to make evidence-informed decisions regarding specialty choice, and thus, the cycle persists [20]. Medical training is an arduous, lengthy, and costly process that can—and should—be optimized by a national PRP strategy.

Currently, there are two main checkpoints in the medical training process that affect the number, mix, and distribution of future physicians, thus acting as a component of national PRP strategy. First, undergraduate medicine admissions; second, postgraduate residency admissions.

### PRP via admissions into medical school

In Canada, medical school admissions represent the number of physicians who will be eligible to practice medicine within the next decade—barring minor exceptions such as visa trainees, immigration or emigration of graduated students, and attrition from medical training. The decision to add or remove these positions is a result of complex processes between the provincial Ministry of Health and their health authorities, the faculties of medicine, and the respective provincial and territorial medical association [21]. Some provinces, such as Québec, alter the number of admissions annually, whereas others can remain unchanged for multiple years [22]. Medical school admissions therefore represents a strategy to plan for the future number of physicians entering the workforce.

The medical school admissions process can also affect the physician workforce composition in two ways. First, individuals originally from a rural region are more likely to stay and practice medicine in a rural region [23–25]. Following this concept, the Northern Ontario School of Medicine (NOSM) was established to train students from Northern Ontario to respond to the region’s need for physicians in rural, Francophone, and Indigenous communities [26]. The school was established in 2005, and consistently above 90% of each incoming class are individuals from Northern Ontario, with an average of 94% staying in Northern Ontario for their professional practice in 2020 [27]. Several other institutions have similar admissions pathways for individuals from underserved or rural communities [28].

Secondly, individuals who share similarities with a particular demographic tend to practice and stay in these communities [23]. Therefore, quotas, coefficients and/or criteria for applicants meeting certain sociodemographic characteristics have been applied in many Canadian medical faculties, and are further highlighted in The Community of Support, a recent partnered initiative from the University of Toronto [29–31]. All 17 Canadian medical faculties now ensure that a minimum number of Indigenous students are admitted [32]. Improving access to high-school and undergraduate mentorship is also an essential component to many of these initiatives. There is presently a paucity of data for the admissions outcomes of such initiatives due to their recency [33]. The overarching goal of such initiatives is to restructure the composition of our physician workforce to better reflect and serve the Canadian population.

The checkpoint of medical school admissions has an influence on PRP in regard to determining the anticipated number of physicians, as well as their distribution by utilising quotas and geographical location of training sites. There are currently no mechanisms in place at this level that have been shown to impact specialty choice. Canadian medical students, therefore, begin medical school with the possibility of pursuing a residency in any discipline offered as part of the Canadian Resident Matching Service (CaRMS).

### PRP via admissions to residency

The CaRMS is a service that aims to match medical students to Canadian residency training positions [34]. The service centralizes all available Canadian residency training positions into one portal. Once a student is matched to a residency program via the CaRMS, they are legally bound to that program, and a minority will also be bound to a return-of-service contract in a predetermined location [35] Overall, the CaRMS accurately depicts residency admissions by demonstrating the quota of residency positions and mix of specialties required by the Canadian population.

While PRP via residency and medical school admissions have helped to improve physician-based HHR in Canada, there remain critical gaps in this approach. The most recent example is the significant rise in the number of unmatched Canadian medical graduates (CMG), reaching a peak of 169 in 2018 after both iterations of the match, and equating to $43.9 M of unrealized public taxpayer investment [18, 36, 37]. These trainees may re-apply for residency in the following year and in the meantime purse a fifth medical school year, another post-graduate degree, or leave medicine altogether. To help balance this gap, $23 million was disbursed over 6 years for new positions in Ontario, and 25 new spots at Dalhousie University were funded by the Nova Scotia Health Ministry [37, 38].

While the unmatched CMG phenomenon is multifactorial, a factual observation is that certain specialties are more competitive than others, as represented by a lower number in the ratio of positions to applicants’ first choice discipline, which correlates with medical student interests. In the 2020 residency match, the most competitive specialty was ophthalmology with a ratio of 0.51, and family medicine was the sixth least competitive specialty with a ratio of 1.65 [39]. Gross income, prestige, and academic opportunities are some factors shown to influence specialty choice [17].. Importantly, competitive specialties are not necessarily those that correlate to the highest population demand, which currently are the specialties of family medicine and geriatrics [13, 40]. In addition, 2 years after the unmatched CMG peak and the subsequent addition of new family medicine positions, the interests of medical students have remained largely unchanged [15].

Altogether, it could be argued that the strategy to add residency positions after the unmatched CMG peak in 2018 imparted a positive impact on the match statistics, but contributed little in terms of addressing the PRP challenges of specialty mix and distribution. Therefore, there is a need to reimagine new PRP strategies after the checkpoint of admission into medical school but prior to participating in the CaRMS match. The goal is to cultivate an interest among medical trainees to choose a specialty which aligns with the quota depicted by the CaRMS checkpoint for admissions to residency. To the best of our knowledge, there are no national approaches to PRP targeting Canadian undergraduate medical trainees to support them in making evidence-informed decisions regarding their specialty choice to match societal demand.

The *HHR Platform* is a first-of-its-kind comprehensive career planning resource for medical students in Canada. It is a national, interactive, web-based tool that uses a map, comparison tables, and trend graphs to illustrate the most relevant public data on current and projected physician workforces across Canada. Our tool empowers future physicians with the technology and data to make evidence-informed decisions regarding specialty choice. This work was undertaken by the Canadian Federation of Medical Students’ (CFMS) HHR Task Force with the goal of shifting the decision-making process for Canadian medical trainees towards both personal interests and social accountability. Over time, these decisions comprise a component of national PRP strategy to help correct the physician supply-demand mismatch across Canada.

## Implementation

The *HHR Platform* was created by FireNet Designs (FireNet Designs, Winnipeg, MB, CA) on a DigitalOcean (DigitalOcean Inc., New York City, NY, US) server to host the backend database using MySQL (Oracle Corp., Redwood Shores, CA, US). Construction of the platform spanned from November 2019 to September 2020. The backend is composed of a comma separated value (CSV) parser application used for adding data to the database, and flexible application programming interface (API) endpoints for querying data. The frontend is constructed using React (Facebook Inc., Menlo Park, CA, US) to manage user functionality and Material UI (Material-UI SAS, Paris, FR) to simplify data processing. Lastly, Leaflet (Vladimir Agafonkin, Kyiv, UA) and Google Charts (Google LLC, Menlo Park, CA, US) are utilized for the map and graph views, respectively. Geographic representation was aided by a public API acquired from the Environmental Systems Research Institute (ESRI) (ESRI, Redlands, CA, USA). The HHR Platform is hosted publicly and free-of-cost on the CFMS website [41].

The *HHR Platform* coalesces data from the following Canadian agencies: Canadian Institute for Health Information (CIHI), Canadian Medical Association (CMA), Canadian Post-MD Education Registry (CAPER), CaRMS, Ontario Medical Students’ Association (OMSA), and ESRI. Appropriate permissions and data-sharing contracts were obtained prior to construction of the platform. The unique datasets obtained from these agencies are categorized in Table 1. The delimiters included in the *HHR Platform* are categorized in Appendix 1. Program directors—who are responsible for guiding the selection and training of postgraduate resident physicians—were contacted to offer their expert opinion on PRP in their specialty and region given their unique position to comment on medical students’ interest in their program, postgraduate training logistics, and job availability in their specialty.. Aside from Ontario, program directors of all Canadian residency programs were identified via the CaRMS and Royal College websites and contacted via a standardized email template (Appendix 2) for their feedback regarding physician needs pertaining to their specialty and region of practice. Program director responses from Ontario in 2019 had already been collected by OMSA and were accordingly shared with the CFMS. Consent was obtained to share their de-identified responses publicly. No altering of responses were conducted and there was no text analysis; all data were subsequently posted as received onto the HHR platform. Members of the HHR Task Force—comprising nine medical students from across Canada—also performed a standardized search on the public domain for specialty-specific resources, which were amalgamated and included as part of the *HHR Platform*. Google analytics (Alphabet Inc., Mountain View, CA, USA) was integrated to demonstrate the utilization of the HHR platform in the first 4 months following the day of its official launch. An issue tracker using GitHub (Microsoft Corp., San Francisco, CA, USA) is available for user feedback on the CFMS website.

**Table 1:** Data points obtained from CIHI, CMA, CAPER, CaRMS, OMSA, and ESRI

Agency	Data obtained
Canadian Institute for Health Information (CIHI)	Number of working physicians Physician to 100,000 population ratios Physicians by age groups Gross wage Number and percentage of rural and urban physicians Number and percentage of male and female physicians
Canadian Medical Association (CMA)	Number of vacancies for each province Number of working physicians Physicians by age group Number of male and female
Canadian Post-MD Education Registry (CAPER)	Number of residents exits per year Number of fellow exits per year Percentage of residents pursuing fellowship training Number of physicians working in province 2 years after having graduated in the same province
Canadian Resident Matching Service (CaRMS)	Number of CMG seats (school-specific) Number of CMG distinct applicants (Canadian region-specific) Number of CMG applicants who ranked discipline as first choice (Canadian region-specific)
Ontario Medical Students’ Association (OMSA)	All Ontario program director comments
Environmental Systems Research Institute (ESRI)	Hospitals map layer 2016 population census map layer

Sustainability was a key consideration in determining the backend database architecture. Thus, we prioritized construction of the database to be flexible such that new datasets may be added on an annual basis and that complex datasets may be promptly queried. Data presented in the platform was unmanipulated with one exception: data were averaged if the same metric was provided by multiple separate sources; averaged data were indicated by parentheses next to the values to indicate the number of sources contributing to the presented data. The range of the data is available in the graph view. Additionally, per CMA privacy policy, cells with fewer than five data points were suppressed. Specialties on the *HHR Platform* are defined per CIHI’s catalogue. Specialities represented in the platform are shown in Appendix 1. Jurisdictions are computed based on provincial boundaries and further subclassified by regional health authorities. Further information regarding data construction and considerations may be found in the *HHR Platform* user guide, which supplements the web tool [42].

## Results

The *HHR Platform* includes information and resources for 62 specialties with over 500,000 unique numerical data points obtained from 37 datasets. Data summarized in the *HHR Platform* is from 2015 onwards, with succeeding data added on an annual basis. There were 96 program director comments in 2019, representing 24.6% of the total individuals contacted. In the first 4 months (September 20, 2020, to January 20, 2021) of the *HHR Platform* being hosted publicly on the CFMS website, there were an average of 20.0 users per day with a total of 2434 different users, of whom 985 returned to the platform. This comprised 24.8% of all CFMS homepage views and 2.9% of all total site pageviews. 95.5% of users visited the platform from within Canada, with the majority visiting from Edmonton (10.3%), Toronto (8.7%), Ottawa (8.3%) and Calgary (6.1%). Assuming that these Canadian users were all medical students, this represents approximately 28% of CFMS’ membership, which includes 15 out of the 17 medical faculties in Canada. The average time spent on the platform was 3 min. The specific actions of users while using the platform, known as event tracking, was not available and this is further described in the limitations section. The HHR Platform was designed to present data in a customized fashion, whereby users select their preferred view between a map, table, or graph view. Each view utilises the same database but provides a unique purpose based on user needs. No feedback or issues were submitted on the issue tracker regarding the HHR platform since its implementation.

The following results section presents the different parts of the HHR platform, which was the primary outcome of this project. The software code can be found in the supplementary materials, with copyright details listed.

### Table view

**Fig. 1:**
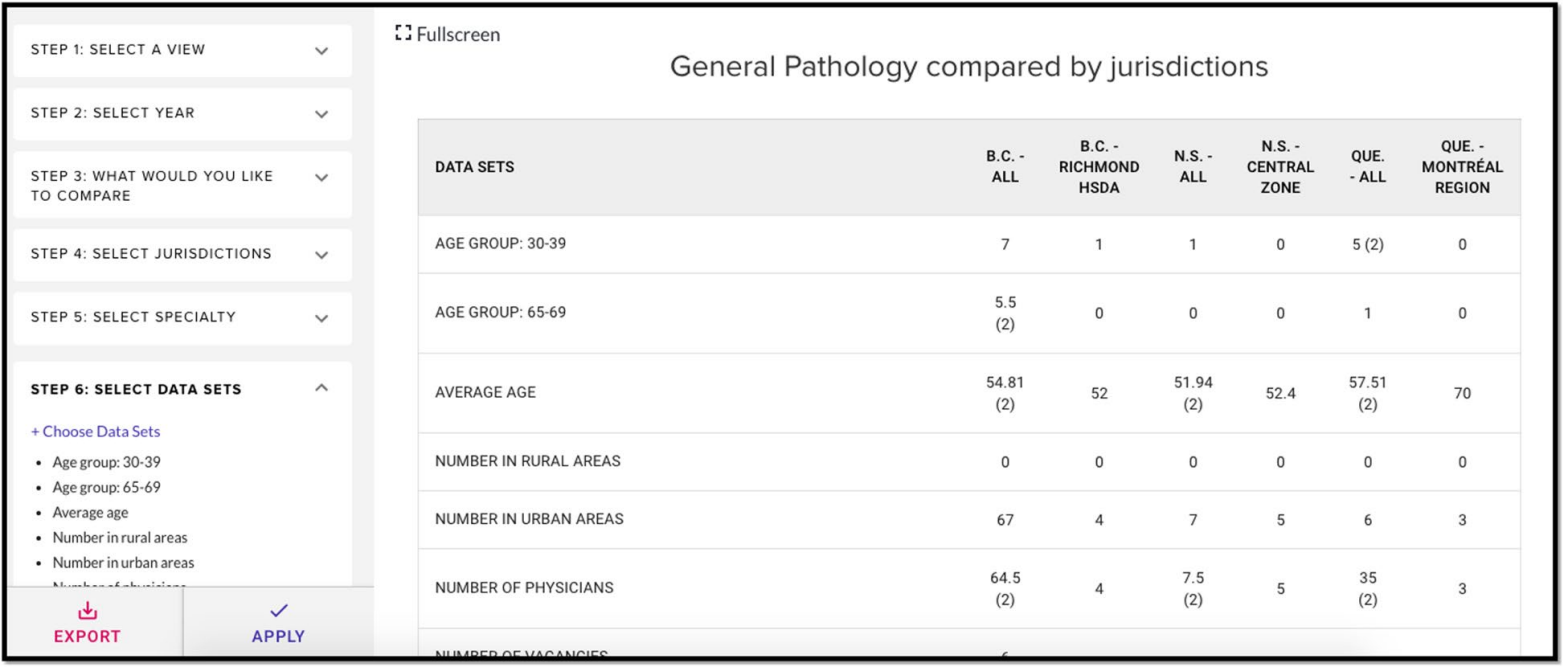
Table view from the *HHR Platform* showing general pathology compared between selected jurisdictions. The authors have the rights to the images depicted in this figure

Table view allows for comparative visualization of granular data (Fig. 1). “Step 1” allows the user to select their preferred view. In “Step 2,” the user selects the year for which wish to access data. “Step 3” allows the user to decide whether they wish to compare multiple specialties across one jurisdiction (‘compare specialty’), or multiple jurisdictions for one specialty (‘compare jurisdiction’). In “Step 4” and “Step 5,” the user selects their jurisdiction(s) and specialty (ies) of interest, respectively. “Step 6” allows the user to choose between the various available datasets.

### Graph view

**Fig. 2:**
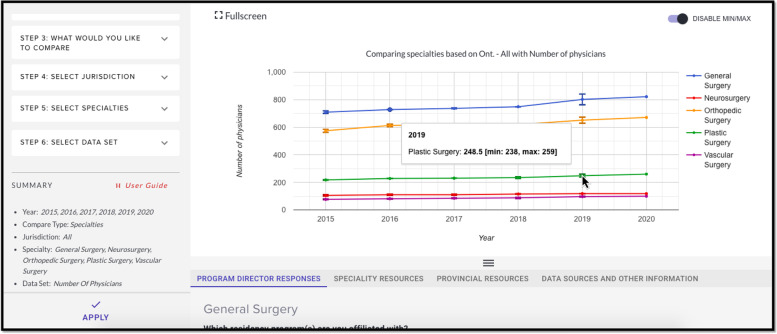
Graph view from *HHR Platform* showing number of physicians for various surgical specialties. The authors have the rights to the images depicted in this figure

Graph view allows for visualization of trends (Fig. 2). This view uses the same steps as above for the table view. A single dataset is chosen over a selected range of time. Hovering over the error bars demonstrate the range of the data coming from the different sources.

### Map view

**Fig. 3:**
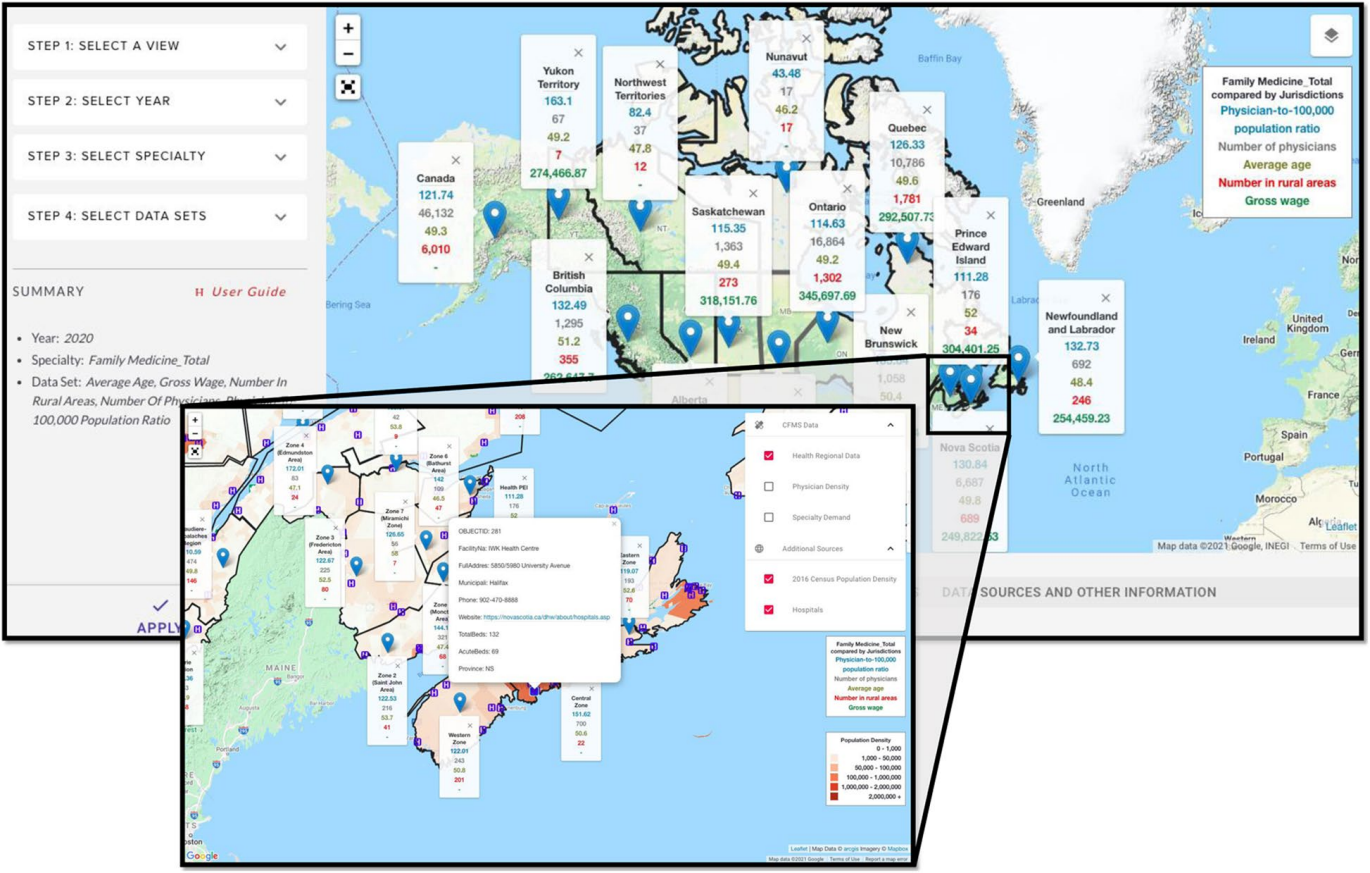
Map view from the *HHR Platform*, with a magnified view showing layers for family medicine. The authors have the rights to the images depicted in this figure

Map view allows for interactive visualization of data across health regions (Fig. 3). The user can compare one specialty across the country for a maximum of five datasets at a time, and the map will refresh as the user interacts with the different zoom levels and geographical locations within the map. Layers can also be superimposed, for example, Canadian hospitals and population density as shown in Fig. 3.

### Useful resources tabs

**Fig. 4:**
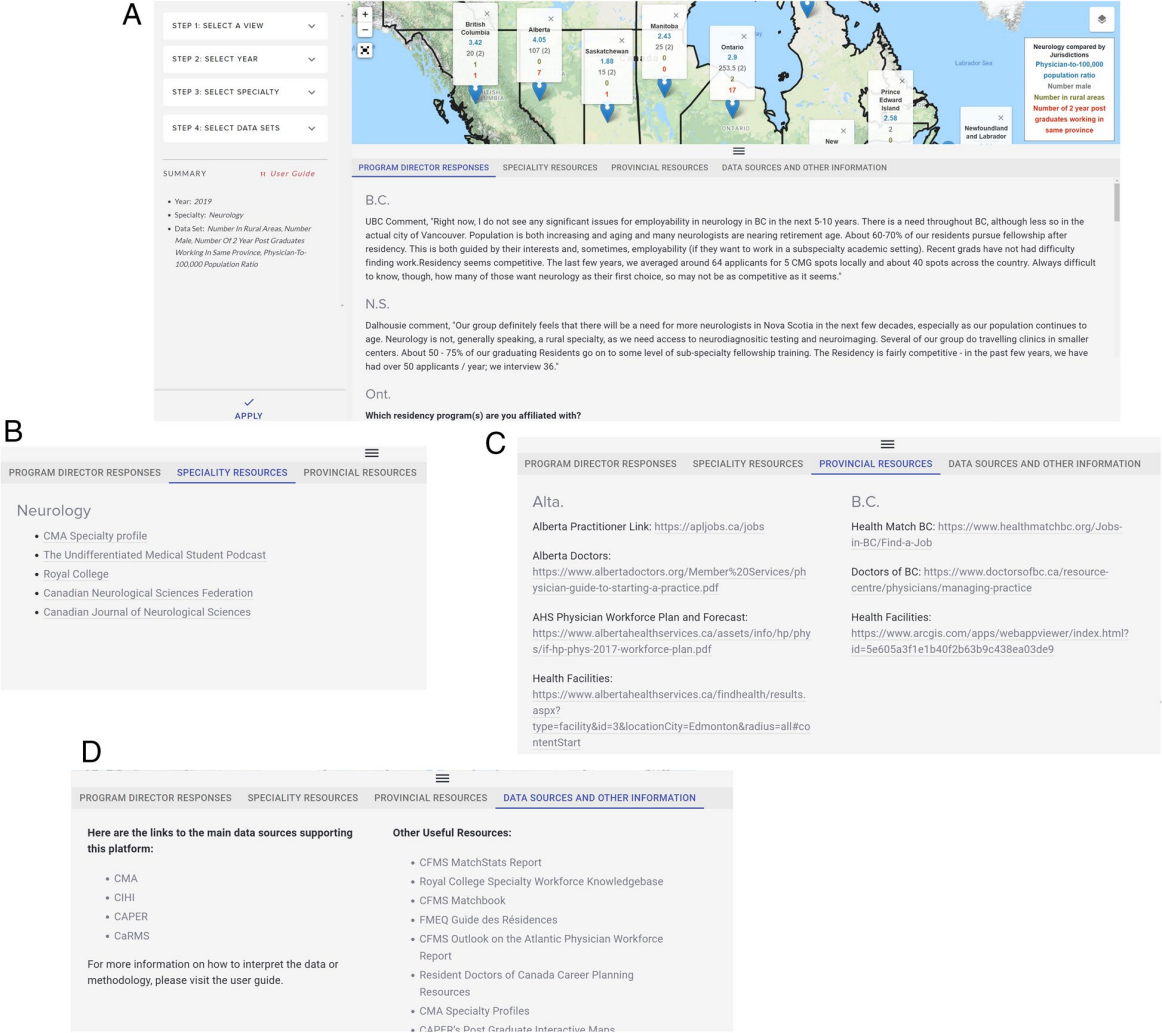
Useful resources of *HHR Platform.* The authors have the rights to the images depicted in this figure. Program director responses (**A**), specialty resources (**B**), provincial resources (**C**), and data sources and other information (**D**) for neurology in 2019. The authors have the rights to the images depicted in this figure

Below the data window are four tabs with additional information that are specific depending on the information queried (Fig. 4). The first tab displays available program director responses specific to the year, specialty, and jurisdiction selected by the user. The second tab displays available specialty resources specific to the selected specialty. The third tab displays available provincial resources. The fourth tab displays data sources and other useful information, such as tools that may provide support in interpreting the data or other helpful resources for medical trainees.

## Discussion

In Canada, the majority of national PRP strategies aimed at the medical training process rely on two checkpoints: admissions into medical school, and admissions into a residency program. While these approaches have shown to be effective in both planning for the number of future physicians entering the workforce and attaining a set specialty mix, the distribution of physician to areas of high needs remains an issue in Canada. Additionally, information and data specifically intended for undergraduate trainees regarding the current and projected demand for physicians in specialties and locations of high population need are lacking [43]. Trainees advance in their medical education by basing their specialty choice on factors such as personal interest, academic opportunities, gross income, and prestige while often not considering social accountability [16, 17]. New strategies that go beyond the admissions checkpoints are warranted to inform trainees of societal needs so that they are inclined to work in areas of high need, ultimately providing better access of care to the Canadian population.

One example of an approach outside of the admissions checkpoint to inform trainees of the population needs was developed by the Fédération Médicale Étudiante du Québec (FMEQ) in 2018. In order to balance physician distribution, the Québec Health Ministry’s rearranged their available residency positions to reflect 55% of seats reserved for the family medicine specialty, and 45% to all other specialties [44]. In that year alone, there were 65 vacant positions following both iterations of the CaRMS in Québec family medicine programs [45, 46]. In response, the FMEQ promoted family medicine education under the pretense that disinterest in family medicine stems from the medical students’ lack of knowledge about this specialty [47]. In the following year in Québec, there was a greater interest in family medicine as demonstrated by an increase from 373 to 440 applicants and only 23 vacancies in family medicine after the first iteration (of two) of the CaRMS match [45, 48]. The FMEQ approached PRP by targeting and educating medical students about the mix and demand of specialties, particularly with respect to the need for family physicians in the Québec population.

Additionally, there are many public resources that play a role in informing Canadian medical trainees on job opportunities and of the different specialties (many of which are outlined in Table 1 and accessible from the HHR Platform) to guide trainees in their career orientation. One limitation of these resources is their focus on a particular dataset or aspect of PRP. Thus, it is difficult for the user to compare these in relation to the data and information they may receive from career advisors, web resources, media reports, or health ministry statements. Another limitation is the frequency at which these various sources share their datasets; most being shared annually with a delay of many months between collection and public availability. In a healthcare system that is vulnerable to change in response to many types of factors (e.g. a pandemic), data must be updated frequently – if not in real-time – for it to remain pertinent. Finally, despite these efforts, strategies aimed at informing medical students’ specialty choice based on predicted population needs remains largely unexploited from a PRP strategy perspective.

The *HHR Platform* represents a national PRP approach towards improving transparency and ease of access to data regarding population health needs and associated practice opportunities to guide medical student specialty choice to be concordant with population health needs. The CFMS has approximately 8300 active medical student members across Canada. With over 2400 different users in the first 4 months of the *HHR Platform* launch, and over 20 daily users on average, there is significant outreach and interest shown by aspiring physicians. Designed with a flexible back-end, the *HHR Platform* is able to integrate new, changing or even incomplete datasets. This will enable it to become increasingly comprehensive over time, therefore functioning as a reliable and up-to-date – potentially even real-time – Canadian resource for career planning among medical trainees. Future directions include incorporating subspecialty data, additional information about workforce composition and demographics, predictive modelling of physician supply-demand dynamics, and inclusion of allied health professionals into the *HHR Platform*. Future analyses may be performed to explore how the *HHR Platform* and population-needs data specifically inform medical student specialty decision-making. The *HHR Platform* is currently equipped for and awaiting datasets for the projected demand of specialties in 5 and/or 10 years. This is likely the most anticipated and relevant dataset to inform medical students of societal needs prior to choosing a specialty, and continued advocacy for its public availability is warranted [49].

### Limitations

The primary limitation is that the HHR Platform depends on continued public release and sharing of data by the collaborating organizations in a format that is compatible with their previous releases. The data amalgamated in the *HHR Platform* is limited by the inclusion and exclusion criteria of the original datasets, which does not allow for exact comparability. For example, the collection of the datasets are undertaken at different points in time depending on the organization, while in the *HHR Platform*, they are grouped per annum. Additionally, we did not receive comments from approximately 75% of program directors. Therefore, there was limited data for some disciplines if students were interested in contextualizing the available quantitative data with qualitative data. To address this limitation, our group plans to contact National Specialty Societies in addition to using a standardized tool to collect program directors’ data longitudinally, and iteratively incorporate these responses to the HHR Platform. Furthermore, the datasets that are available nationally are a result of provincial data collection and sharing, which is not standardized between provinces, and is inconsistent across the various population demographics within Canada [50]. A Canadian common data standard – defined as a set of rules to follow during data collection, processing and reporting – would be a first step [51]. The lack of detailed user usage data and their feedback of the *HHR Platform* is a further limitation. While information relating to number of users was available from Google Analytics, user event tracking functionality was not implemented at the time of data collection. These behaviours would be of relevance to health administration in choosing their strategy for physician retention and recruitment for specialties that are less desirable but in high need. The System Usability Scale (SUS) will be implemented on the next version of the platform to obtain user feedback [52].

## Conclusion

There is mounting evidence that Canadian medical students have limited information and data on the determinants of PRP, thereby lacking the tools to inform their specialty decision to be socially accountable based on population needs [53, 54]. The purpose of the *HHR Platform* is to provide a national approach to PRP which informs undergraduate medical trainees on the number, mix, and distribution of physicians to meet population health needs of Canadians. Modernising health data delivery by depicting the workforce needs of the population to medical students prior to making their specialty choice may function to improve the utilisation of current human resources, thus alleviating the impending shortage of health workers. Given the worldwide physician shortage, such a platform may be modelled in other countries with the goal of optimizing their physician workforce composition to better align with the needs of their population.

### Availability and requirements

Project name: CFMS Health Human Resources Platform.

Project home page; https://www.cfms.org/; https://www.cfms.org/resources/; https://www.cfms.org/resources/health-human-resources-platform/

Operating system(s): Platform independent.

Programming language: MySQL (C and C++), React and Material UI (JavaScript).

Other requirements: No other requirements.

License: N/A.

Any restrictions to use by non-academics: No restrictions.

## Data Availability

The data generated or mentioned in study are included on the *HHR platform* publicly available at https://www.cfms.org/resources/health-human-resources-platform/. As of March 5th 2021, the datasets were available in the CIHI [55], CAPER [56], CaRMS [57] and CMA [58] registries. The platform as a service with all data removed, is available in the supplementary file. For the datasets regarding website activity, they are available from the corresponding author on reasonable request.

## Appendix 1

Table 2

**Table 2:** Datasets generated from CIHI, CMA, CAPER, CaRMS, OMSA, and ESRI

Dataset	Class	Subclass
*Specialties*	*Non-surgical disciplines*	Anatomical pathology
		Anesthesiology
		Cardiology
		Clinical immunology and allergy
		Clinical pharmacology and toxicology
		Critical care medicine
		Dermatology
		Diagnostic radiology
		Emergency medicine
		Endocrinology and metabolism
		Family medicine - Care of the elderly
		Family medicine - Emergency
		Family medicine - General practice
		Family medicine - Palliative care
		Family medicine - Total
		Gastroenterology
		General internal medicine
		General pathology
		Geriatrics
		Hematology
		Infectious diseases
		Medical genetics
		Medical microbiology
		Medical oncology
		Nephrology
		Neuropathology
		Nuclear medicine
		Pain medicine
		Palliative medicine
		Pediatrics - Adolescent medicine
		Pediatrics - Clinical immunology and allergy
		Pediatrics - Developmental
		Pediatrics - Emergency medicine
		Pediatrics - Endocrinology and metabolism
		Pediatrics - Gastroenterology
		Pediatrics - General
		Pediatrics - Hematology-oncology
		Pediatrics - Infectious diseases
		Pediatrics - Neonatal-perinatal medicine
		Pediatrics - Nephrology
		Pediatrics - Neurology
		Pediatrics - Respirology
		Physical medicine and rehabilitation
		Psychiatry
		Public health and preventive medicine
		Radiation oncology
		Rheumatology
	*Surgical disciplines*	Cardiac and thoracic surgery
		Neurosurgery
		Nuclear medicine
		Obstetrics and gynecology
		Ophthalmology
		Orthopedic surgery
		Otolaryngology - Head and neck surgery
		Plastic surgery
		Urology
		Vascular surgery
*Jurisdictions*	**Provinces and territories**	**Health regions**
	*Newfoundland and Labrador*	Eastern Health
		Central Health
		Western Health
		Labrador–Grenfell Health
	*Prince Edward Island*	Health PEI
	*Nova Scotia*	Western Zone
		Northern Zone
		Eastern Zone
		Central Zone
	*New Brunswick*	Zone 1 (Moncton Area)
		Zone 2 (Saint John Area)
		Zone 3 (Fredericton Area)
		Zone 4 (Edmundston Area)
		Zone 5 (Campbellton Area)
		Zone 6 (Bathurst Area)
		Zone 7 (Miramichi Zone)
	*Québec*	Bas-Saint-Laurent Region
		Saguenay–Lac-Saint-Jean Region
		Capitale-Nationale Region
		Mauricie et Centre-du-Québec Region
		Estrie Region
		Montréal Region
		Outaouais Region
		Abitibi-Témiscamingue Region
		Côte-Nord Region
		Nord-du-Québec Region
		Gaspésie–Îles-de-la-Madeleine Region
		Chaudière-Appalaches Region
		Laval Region
		Lanaudière Region
		Montérégie Region
		Nunavik Region
		Terre-Cries-de-la-Baie-James Region
	*Ontario*	Erie St. Clair LHIN
		South West LHIN
		Waterloo Wellington LHIN
		Hamilton Niagara Haldimand Brant LHIN
		Central West LHIN
		Mississauga Halton LHIN
		Toronto Central LHIN
		Central LHIN
		Central East LHIN
		South East LHIN
		Champlain LHIN
		North Simcoe Muskoka LHIN
		North East LHIN
		North West LHIN
	*Manitoba*	Winnipeg Regional Health Authority
		Prairie Mountain Health
		Interlake–Eastern Regional Health Authority
		Northern Health Region
		Southern Health — Santé Sud
		*Saskatchewan*
		Sun Country Health Region
		Five Hills Health Region
		Cypress Health Region
		Regina Qu’Appelle Health Region
		Sunrise Health Region
		Saskatoon Health Region
		Heartland Health Region
		Kelsey Trail Health Region
		Prince Albert Parkland Health Region
		Prairie North Health Region
		Mamawetan Churchill River Health Region
		Keewatin Yatthé Health Region
		Athabasca Health Authority
	*Alberta*	South Zone
		Calgary Zone
		Central Zone
		Edmonton Zone
		North Zone
	*British Columbia*	East Kootenay HSDA
		Kootenay–Boundary HSDA
		Okanagan HSDA
		Thompson/Cariboo HSDA
		Fraser East HSDA
		Fraser North HSDA
		Fraser South HSDA
		Richmond HSDA
		Vancouver HSDA
		North Shore/Coast Garibaldi HSDA
		South Vancouver Island HSDA
		Central Vancouver Island HSDA
		North Vancouver Island HSDA
		Northwest HSDA
		Northern Interior HSDA
		Northeast HSDA
	*Yukon Territory*	Yukon Territory
	*Northwest Territory*	Northwest Territory
	*Nunavut*	Nunavut
*Data sets*	*Gender demographics*	Number of physicians
		Number male
		Number female
		Number sex unknown
		Percentage female
		Percentage male
	*Age demographics*	Average age
		Median age
		Age group: younger than 30
		Age group: 30-39
		Age group: 40-49
		Age group: 50-59
		Age group: 60-64
		Age group: 65-69
		Age group: 70-74
		Age group: 75-79
		Age group: 80 and older
		Age group: Unknown
	*Location distribution*	Number in rural areas
		Number in urban areas
		Number unknown urban or rural
		Percentage rural
		Percentage urban
	*Training metrics*	Number of CMG seats
		Number of CMG applicants
		Number of CMG applicants who ranked specialty as first choice
		Number of 2 year post graduates working in same province
		Number of resident exits per year
		Number of fellow exits per year
		Percentage of residents pursuing fellowship training
		Ratio of number of CMG applicants who ranked specialty as first choice to number of CMG seats
	*Employment metrics*	Number of vacancies
		Number of graduates from province 2 years ago
		Ratio of number of graduates from province 2 years ago to number of 2 year post graduates working in same province
		Ratio of number of vacancies to number of physicians
		5 year projected need
		10 year projected need
		Physician-to-100,000 population ratio
		Gross wage
	*Additional resources*	Program director responses
		Specialty resources
		Provincial resources

## Appendix 2

Program director letter for specialty commentaries

## Abbreviations

HHR: Health Human Resource
PRP: Physician Resource Planning
CFMS: Canadian Federation of Medical Students
CSV: Comma Separated Value
API: Application Programming Interface
ESRI: Environmental Systems Research Institute
CIHI: Canadian Institute for Health Information
CMA: Canadian Medical Association
CAPER: Canadian Post-MD Education Registry
CaRMS: Canadian Resident Matching Service
OMSA: Ontario Medical Students Association
NOSM: Northern Ontario School of Medicine
CMG: Canadian Medical Graduates
FMEQ: Fédération Médicale Étudiante du Québec
